# A dimer of bis­(N-heterocyclic carbene)rhodium(I) centres spanned by a dibenzo-18-crown-6 bridge from synchrotron radiation

**DOI:** 10.1107/S160053681204901X

**Published:** 2012-12-12

**Authors:** Sumi Shrestha, Mohan Bhadbhade, Carolina Gimbert-Suriñach, Stephen B. Colbran

**Affiliations:** a School of Chemistry, University of New South Wales, Sydney, NSW 2052, Australia; b Mark Wainwright Analytical Centre, University of New South Wales, Sydney, NSW 2052, Australia

## Abstract

The compound (μ-3,3′,3′′,3′′′-{[2,5,8,15,18,21-hexa­oxatricyclo­[20.4.0.0^9,14^]hexa­cosa-1(22),9,11,13,23,25-hexa­ene-11,12,24,25-tetra­yl]tetra­kis­(methyl­ene)}tetra­kis­(1-methyl-1*H*-imidazol-2-yl))bis­[(η^4^-cyclo­octa-1,4-diene)rhodium(I)] bis­(hexa­fluoridophosphate) acetonitrile sesquisolvate dihydrate, [Rh_2_(C_8_H_12_)_2_(C_40_H_42_N_8_O_6_)](PF_6_)_2_·1.5CH_3_CN·2H_2_O, crystallized from acetonitrile under an atmosphere of diethyl ether. In the crystal structure, the complex cation exhibits two square-planar Rh^I^ centres, each bound by a cyclo­octa­diene (COD) ligand and by two adjacent imidazolyl­idene N-heterocyclic carbene (NHC) donors from the same phen­oxy ring of the {[dibenzo-18-crown-6-11,12,24,25-tetra­yl]tetra­kis­(methyl­ene)}tetra­kis­(1-methyl-1*H*-imidazol-2-yl) (*L*) ligand. The dibenzo-crown ether bridge of *L* spans the Rh centres and forms hydrogen bonds with water mol­ecules. One water mol­ecule with half occupancy bridges adjacent macrocycles in the lattice. Another water with full occupancy forms weak hydrogen bonds to the crown ether O atoms and is, in turn, part hydrogen bonded by a lattice water with half occupancy. The latter water is within hydrogen-bonding distance of a fourth water also with partial occupancy. The result of these inter­actions is the formation of a layer in the *ab* plane. Two PF_6_^−^ ions, one of which is twofold disordered, and one ordered and one twofold disordered (with 0.5 occupancy) lattice acetonitrile mol­ecules complete the crystal structure.

## Related literature

For the related complex [K(*L*){Rh(COD)}_2_][PF_6_]_3_, which has a potassium ion bound within the crown ether bridge of the ligand *L*, see: Shrestha *et al.* (2011 ▶). For the well known Rh(I)(NHC)_2_(COD) centres, see: Mata *et al.* (2004 ▶); Riederer *et al.* (2010 ▶).
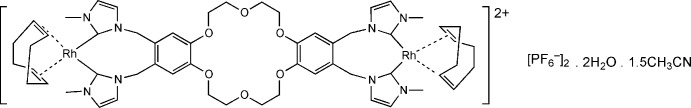


## Experimental

### 

#### Crystal data

[Rh_2_(C_8_H_12_)_2_(C_40_H_42_N_8_O_6_)](PF_6_)_2_·1.5C_2_H_3_N·2H_2_O
*M* *_r_* = 1540.55
Triclinic, 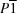

*a* = 10.510 (2) Å
*b* = 15.630 (3) Å
*c* = 23.280 (5) Å
α = 104.69 (3)°
β = 90.20 (3)°
γ = 109.58 (3)°
*V* = 3468.9 (12) Å^3^
*Z* = 2
Synchrotron radiation
λ = 0.71073 Å
μ = 0.61 mm^−1^
*T* = 100 K
0.03 × 0.02 × 0.01 mm

#### Data collection

3-BM1 Australian Synchrotron diffractometer
43942 measured reflections
11473 independent reflections
9939 reflections with *I* > 2σ(*I*)
*R*_int_ = 0.038

#### Refinement

*R*[*F* ^2^ > 2σ(*F* ^2^)] = 0.051
*wR*(*F* ^2^) = 0.164
*S* = 1.33
11473 reflections
964 parameters
270 restraints
H atoms treated by a mixture of independent and constrained refinement
Δρ_max_ = 1.15 e Å^−3^
Δρ_min_ = −1.40 e Å^−3^

### 

Data collection: *BLU-ICE* (McPhillips *et al.*, 2002 ▶); cell refinement: *XDS* (Kabsch, 1993 ▶); data reduction: *XDS*; program(s) used to solve structure: *SHELXS97* (Sheldrick, 2008 ▶) and *OLEX2* (Dolomanov *et al.*, 2009 ▶); program(s) used to refine structure: *SHELXL97* (Sheldrick, 2008 ▶); molecular graphics: *ORTEP-3 for Windows* (Farrugia, 2012 ▶); software used to prepare material for publication: *publCIF* (Westrip, 2010 ▶).

## Supplementary Material

Crystal structure: contains datablock(s) I, global. DOI: 10.1107/S160053681204901X/tk5170sup1.cif

Structure factors: contains datablock(s) I. DOI: 10.1107/S160053681204901X/tk5170Isup2.hkl

Additional supplementary materials:  crystallographic information; 3D view; checkCIF report

## Figures and Tables

**Table 1:** Hydrogen-bond geometry (Å, °)

*D*—H⋯*A*	*D*—H	H⋯*A*	*D*⋯*A*	*D*—H⋯*A*
O1*M*—H1*MB*⋯O2^i^	0.86 (1)	2.51 (3)	3.226 (5)	141 (3)
O1*M*—H1*MB*⋯O3^i^	0.86 (1)	2.50 (4)	3.076 (4)	125 (4)
O1*M*—H1*MA*⋯O1^i^	0.86 (1)	2.38 (3)	3.149 (5)	149 (4)
O1*M*—H1*MA*⋯O6^i^	0.86 (1)	2.39 (2)	3.138 (5)	145 (4)
O3*W*—H3*WB*⋯O5^i^	0.87 (1)	2.21 (9)	3.03 (2)	158 (23)
O3*W*—H3*WA*⋯O2^ii^	0.87 (1)	2.16 (10)	2.99 (2)	160 (27)
O1*W*—H1*WA*⋯O1*M*	0.87 (1)	2.00 (1)	2.796 (9)	151 (3)
O1*W*—H1*WB*⋯N1*CN*	0.87 (1)	2.06 (6)	2.805 (18)	143 (9)

Symmetry codes: (i) 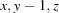; (ii) 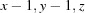.

**Fig. 1.**
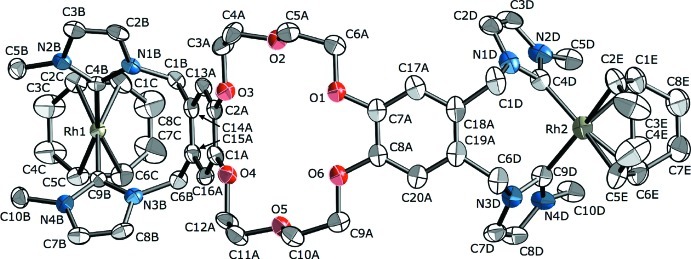
View of the [(L){Rh(COD)}2]2+ cation showing 50% thermal ellipsoids (H-atoms are omitted for clarity).

**Fig. 2.**
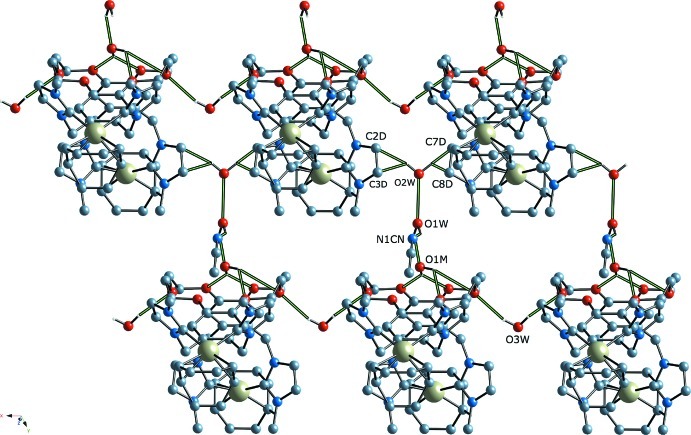
Ball-and-stick view showing the packing of [(L){Rh(COD)}2]2+ cations and lattice water molecules in the crystal structure. The labelling scheme adopted for the waters is shown.

## supplementary crystallographic information

### Comment

The sum of the acute C(NHC)-Rh-{C=C(centroid) for COD} bond angles is 360.2° for Rh1 and 359.3° for Rh2 indicative for the planarity of these centres. The Rh–C bond lengths are in the normal range (Mata *et al.*, 2004; Riederer *et al.*, 2010; Shrestha *et al.*, 2011): 2.033 (4) - 2.042 (4) Å for the Rh—C(NHC) and 2.196 (5) - 2.214 (5) Å for the Rh–C(COD) distances. The dibenzo-18-crown-6 bridge adopts an 'umbrella' shape and forms hydrogen bonds with a centrally located water molecule (O1*M*; see Fig. 2). Other lattice water with partial occupancy are observed in the structure and are depicted in Fig. 2. To use the nomenclature introduced in Shrestha *et al.* (2011), the Rh(NHC)_2_(COD) centres are aligned 'up-and-out' for Rh1 and 'down-and-out' for Rh2.

### Experimental

The synthesis of [(*L*){Rh(COD)}_2_]Br_2_ (*L* = bis{4,5-bis(1-methylene-3-methyl- imidazolidene)}benzo-18-crown-6; COD = 1,5-cyclooctadiene) has been described by us (Shrestha *et al.*, 2011). This was dissolved in methanol and treated with aqueous [NH_4_][PF_6_]. The yellow-orange precipitate was collected by filtration, and recrystallized from acetonitrile under an atmosphere of diethyl ether to afford very thin yellow crystalline platelets of the title complex, which were used for this X-ray crystal structure determination.

### Refinement

The crystal lattice contained one ordered and one orientationally disordered PF_6_^-^ anions and one ordered and one orientationally disordered acetonitrile (solvent) molecules. In addition, there is one lattice water with full occupancy and there are three water molecules with partial occupancies. All the disorders were modelled keeping the geometries of each entity in question restrained using *DFIX* / SADI commands and the atomic displacement parameters were restrained using DELU / SIMU commands. The H atoms to the water molecules were fixed so as achieve the best possible O—H···O interactions between them. The low occupancy entities were kept isotropic throughout the refinement.

A total of four water molecules have been located in difference Fourier maps, which are at favorable hydrogen bonding distances from each other and other possible H-bonding groups. However, difference Fourier maps did not reveal the H-atoms attached to these waters, probably because of the possible orientational disorder and/or low occupancies of some of the water molecules. One of the possible constellations for water H-atoms is modeled in the present structure using OLEX-2 software (Dolomanov *et al.*, 2009).

Amongst the waters, O1*M* situated at the centre of the crown has the full occupancy. However, it is almost equidistant from the O atoms O1, O2, O3, O4, O5 and O6 of the crown (distances range from 3.060 – 3.219 Å), all of which are larger than the normally observed O···O distances (2.6 – 2.8 Å). Therefore, this water is likely to be orientationally disordered with H-atoms making weaker O—H···O hydrogen bonds with any of the pairs of crown O-atoms or forming bifurcated interactions. In the present model, the hydrogen atoms H1MA and H1MB make bifurcated O—H···O interactions with oxygen atoms O1, O6 and O2, O3 respectively. Water molecule O1W (occupancy 1/2) at a distance of 2.80 Å from O1*M* is modeled to make two H-bonding interactions, one with the water O1*M* (O1W– H1WA···O1*M*) and the other being the O—H···N contact with the major site of an acetonitrile (O1W—H1WB···N1CN). One of the H-atoms on O1W could also make O—H···O contact with the lowest occupied water molecule O2W (occupancy 1/4), but this would leave the acetonitrile without any (binding) short contact. It could be possible that one of the H-atoms on O2W makes O—H···O contact with the water O1W. However, considering the twofold symmetry about this water, it is preferred that both the H-atoms of O2W make O—H···π contacts with the adjacent imidazolylidene rings with shorter approaches to C2D, C3D on one side and C7D, C8D on the other. As O2W has low occupancy, efforts were not made to optimize the O—H···pi contacts. Water molecule O3W, with a partial occupancy (1/2), exhibits obvious O—H···O bonding: it forms O3W—H3WA···O2 and O3W—H3WB···O5 bridges between adjacent crown ether macrocycles in the crystal lattice.

Carbon-bound H-atoms were placed in calculated positions (C—H 0.93 to 0.97 Å) and were included in the refinement in the riding model approximation, with *U*_iso_(H) = 1.2-1.5*U*_equiv_(C).

### Crystal data

**Table:**

[Rh_2_(C_8_H_12_)_2_(C_40_H_42_N_8_O_6_)](PF_6_)_2_·1.5C_2_H_3_N·2H_2_O	*Z* = 2
*M**_r_* = 1540.55	*F*(000) = 1574
Triclinic, *P*1	*D*_x_ = 1.475 Mg m^−^^3^
Hall symbol: -P 1	Synchrotron radiation, λ = 0.71073 Å
*a* = 10.510 (2) Å	Cell parameters from 9980 reflections
*b* = 15.630 (3) Å	θ = 2.5–22.5°
*c* = 23.280 (5) Å	µ = 0.61 mm^−^^1^
α = 104.69 (3)°	*T* = 100 K
β = 90.20 (3)°	Plates, yellow
γ = 109.58 (3)°	0.03 × 0.02 × 0.01 mm
*V* = 3468.9 (12) Å^3^	

### Data collection

**Table:**

3-BM1 Australian Synchrotron diffractometer	9939 reflections with *I* > 2σ(*I*)
Radiation source: Synchrotron BM	*R*_int_ = 0.038
Si<111> monochromator	θ_max_ = 25.0°, θ_min_ = 1.8°
Phi Scan scans	*h* = −12→12
43942 measured reflections	*k* = −18→18
11473 independent reflections	*l* = −27→27

### Refinement

**Table:**

Refinement on *F*^2^	Primary atom site location: structure-invariant direct methods
Least-squares matrix: full	Secondary atom site location: difference Fourier map
*R*[*F*^2^ > 2σ(*F*^2^)] = 0.051	Hydrogen site location: inferred from neighbouring sites
*wR*(*F*^2^) = 0.164	H atoms treated by a mixture of independent and constrained refinement
*S* = 1.33	*w* = 1/[σ^2^(*F*_o_^2^) + (0.1*P*)^2^ + 0.5989*P*] where *P* = (*F*_o_^2^ + 2*F*_c_^2^)/3
11473 reflections	(Δ/σ)_max_ = 0.001
964 parameters	Δρ_max_ = 1.15 e Å^−^^3^
270 restraints	Δρ_min_ = −1.40 e Å^−^^3^

### Special details

**Table:**

Geometry. All e.s.d.'s (except the e.s.d. in the dihedral angle between two l.s. planes) are estimated using the full covariance matrix. The cell e.s.d.'s are taken into account individually in the estimation of e.s.d.'s in distances, angles and torsion angles; correlations between e.s.d.'s in cell parameters are only used when they are defined by crystal symmetry. An approximate (isotropic) treatment of cell e.s.d.'s is used for estimating e.s.d.'s involving l.s. planes.
Refinement. Refinement of *F*^2^ against ALL reflections. The weighted *R*-factor *wR* and goodness of fit *S* are based on *F*^2^, conventional *R*-factors *R* are based on *F*, with *F* set to zero for negative *F*^2^. The threshold expression of *F*^2^ > σ(*F*^2^) is used only for calculating *R*-factors(gt) *etc*. and is not relevant to the choice of reflections for refinement. *R*-factors based on *F*^2^ are statistically about twice as large as those based on *F*, and *R*- factors based on ALL data will be even larger.The crystal lattice contained one ordered and one orientationally disordered PF_6_^-^ anions and one ordered and one orientationally disordered acetonitrile (solvent) molecules. In addition, there is one lattice water with full occupancy and there are three water molecules with partial occupancies. All the disorders were modelled keeping the geometries of each entity in question restrained using *DFIX* / SADI commands and the atomic displacement parameters were restrained using DELU / SIMU commands. The H atoms to the water molecules were fixed so as achieve the best possible O—H···O interactions between them. The low occupancy entities were kept isotropic throughout the refinement.A total of four water molecules have been located in difference Fourier maps, which are at favorable hydrogen bonding distances from each other and other possible H-bonding groups. However, difference Fourier maps did not reveal the H-atoms attached to these waters, most probably because of the possible orientational disorder and/or low occupancies of some of the water molecules. One of the possible constellations for water H-atoms is modeled in the present structure using OLEX-2 v1.2 software (Dolomanov *et al.*, 2009).

### Fractional atomic coordinates and isotropic or equivalent isotropic displacement parameters (Å^2^)

**Table:**

	*x*	*y*	*z*	*U*_iso_*/*U*_eq_	Occ. (<1)
Rh1	0.60101 (3)	1.202962 (19)	−0.134418 (12)	0.02157 (12)	
C1C	0.6876 (5)	1.1042 (3)	−0.1908 (2)	0.0417 (11)	
H1C	0.7331	1.1033	−0.1568	0.050*	
C2C	0.7299 (5)	1.1861 (3)	−0.20809 (17)	0.0353 (10)	
H2C	0.8030	1.2361	−0.1854	0.042*	
C3C	0.6669 (7)	1.2010 (6)	−0.2608 (3)	0.074 (2)	
H3C	0.7160	1.2101	−0.2933	0.089*	
C4C	0.5322 (6)	1.2011 (6)	−0.2610 (3)	0.074 (2)	
H4C	0.4918	1.2102	−0.2935	0.089*	
C5C	0.4553 (4)	1.1865 (3)	−0.20789 (17)	0.0332 (9)	
H5C	0.4334	1.2367	−0.1848	0.040*	
C6C	0.4158 (5)	1.1050 (3)	−0.1915 (2)	0.0417 (11)	
H6C	0.3685	1.1038	−0.1578	0.050*	
C7C	0.4411 (7)	1.0166 (4)	−0.2224 (4)	0.085 (2)	
H7C	0.3691	0.9625	−0.2416	0.102*	
C8C	0.5743 (7)	1.0162 (4)	−0.2224 (4)	0.087 (2)	
H8C	0.5918	0.9620	−0.2417	0.104*	
N1B	0.7968 (3)	1.2112 (2)	−0.03429 (14)	0.0281 (7)	
N2B	0.8752 (3)	1.3339 (2)	−0.06641 (14)	0.0286 (7)	
N3B	0.4146 (3)	1.2115 (2)	−0.03475 (14)	0.0283 (7)	
N4B	0.4581 (3)	1.3342 (2)	−0.06650 (14)	0.0281 (7)	
C1B	0.7069 (4)	1.1216 (3)	−0.02549 (18)	0.0312 (9)	
H1B1	0.7598	1.0822	−0.0227	0.037*	
H1B2	0.6399	1.0887	−0.0597	0.037*	
C2B	0.9229 (4)	1.2639 (3)	−0.00328 (18)	0.0341 (10)	
H2B	0.9651	1.2489	0.0260	0.041*	
C3B	0.9726 (4)	1.3406 (3)	−0.02344 (18)	0.0344 (10)	
H3B	1.0561	1.3888	−0.0111	0.041*	
C4B	0.7667 (4)	1.2541 (3)	−0.07322 (16)	0.0245 (8)	
C5B	0.8905 (5)	1.4033 (3)	−0.0994 (2)	0.0378 (10)	
H5B1	0.8084	1.3867	−0.1245	0.057*	
H5B2	0.9645	1.4049	−0.1236	0.057*	
H5B3	0.9086	1.4643	−0.0720	0.057*	
C6B	0.4144 (4)	1.1213 (3)	−0.02577 (18)	0.0305 (9)	
H6B1	0.4484	1.0883	−0.0598	0.037*	
H6B2	0.3221	1.0821	−0.0232	0.037*	
C7B	0.3685 (4)	1.3410 (3)	−0.02353 (18)	0.0349 (10)	
H7B	0.3338	1.3894	−0.0110	0.042*	
C8B	0.3415 (4)	1.2638 (3)	−0.00349 (18)	0.0335 (9)	
H8B	0.2847	1.2487	0.0257	0.040*	
C9B	0.4873 (4)	1.2543 (3)	−0.07371 (16)	0.0237 (8)	
C10B	0.5125 (5)	1.4037 (3)	−0.0996 (2)	0.0380 (10)	
H10A	0.5735	1.3849	−0.1263	0.057*	
H10B	0.5605	1.4640	−0.0722	0.057*	
H10C	0.4395	1.4083	−0.1221	0.057*	
Rh2	0.30618 (4)	0.61278 (2)	0.415306 (15)	0.03863 (14)	
C1E	0.3928 (6)	0.5140 (4)	0.4374 (3)	0.0533 (13)	
H1E	0.4393	0.5146	0.4035	0.064*	
C2E	0.4338 (6)	0.5957 (4)	0.4838 (2)	0.0486 (12)	
H2E	0.5062	0.6463	0.4786	0.058*	
C3E	0.3717 (8)	0.6099 (7)	0.5419 (3)	0.089 (2)	
H3E1	0.3745	0.5607	0.5596	0.107*	
H3E2	0.4297	0.6694	0.5682	0.107*	
C4E	0.2381 (7)	0.6104 (7)	0.5416 (3)	0.089 (2)	
H4E1	0.2407	0.6701	0.5679	0.107*	
H4E2	0.1856	0.5614	0.5594	0.107*	
C5E	0.1602 (6)	0.5968 (4)	0.4835 (2)	0.0487 (12)	
H5E	0.1369	0.6470	0.4784	0.058*	
C6E	0.1207 (5)	0.5140 (4)	0.4370 (3)	0.0515 (13)	
H6E	0.0759	0.5141	0.4025	0.062*	
C7E	0.1443 (7)	0.4247 (4)	0.4377 (4)	0.094 (3)	
H7E	0.0721	0.3709	0.4387	0.113*	
C8E	0.2791 (7)	0.4240 (4)	0.4370 (5)	0.096 (3)	
H8E	0.2968	0.3693	0.4364	0.115*	
N1D	0.5737 (4)	0.7555 (3)	0.40038 (16)	0.0404 (9)	
N2D	0.5296 (4)	0.6290 (3)	0.32954 (17)	0.0429 (9)	
N3D	0.1805 (4)	0.7558 (3)	0.39959 (17)	0.0397 (9)	
N4D	0.0999 (4)	0.6295 (3)	0.32853 (17)	0.0413 (9)	
C1D	0.5603 (6)	0.8278 (3)	0.45105 (19)	0.0454 (12)	
H1D1	0.6468	0.8593	0.4752	0.054*	
H1D2	0.4946	0.7979	0.4755	0.054*	
C2D	0.6872 (5)	0.7661 (4)	0.3674 (2)	0.0459 (12)	
H2D	0.7668	0.8183	0.3752	0.055*	
C3D	0.6599 (5)	0.6878 (4)	0.3231 (2)	0.0488 (13)	
H3D	0.7157	0.6747	0.2938	0.059*	
C4D	0.4766 (4)	0.6702 (3)	0.37734 (18)	0.0355 (10)	
C5D	0.4610 (5)	0.5341 (4)	0.2892 (2)	0.0503 (13)	
H5D1	0.5012	0.4912	0.2976	0.075*	
H5D2	0.4707	0.5363	0.2486	0.075*	
H5D3	0.3664	0.5130	0.2953	0.075*	
C6D	0.2675 (5)	0.8278 (3)	0.45104 (19)	0.0432 (11)	
H6D1	0.3034	0.7972	0.4751	0.052*	
H6D2	0.2131	0.8593	0.4754	0.052*	
C7D	0.0796 (5)	0.7668 (4)	0.3668 (2)	0.0446 (11)	
H7D	0.0528	0.8193	0.3744	0.053*	
C8D	0.0278 (5)	0.6878 (4)	0.3221 (2)	0.0478 (12)	
H8D	−0.0418	0.6745	0.2929	0.057*	
C9D	0.1938 (5)	0.6706 (3)	0.37649 (19)	0.0363 (10)	
C10D	0.0738 (5)	0.5352 (4)	0.2884 (2)	0.0492 (13)	
H10D	0.1485	0.5148	0.2939	0.074*	
H10E	0.0638	0.5372	0.2478	0.074*	
H10F	−0.0079	0.4918	0.2973	0.074*	
O1	0.6786 (3)	1.1124 (2)	0.37871 (12)	0.0340 (7)	
O2	0.8558 (3)	1.1616 (2)	0.28704 (13)	0.0363 (7)	
O3	0.7223 (3)	1.1987 (2)	0.19369 (12)	0.0344 (7)	
O4	0.4766 (3)	1.1983 (2)	0.19345 (12)	0.0335 (6)	
O5	0.3066 (3)	1.1615 (2)	0.28660 (12)	0.0369 (7)	
O6	0.4340 (3)	1.1127 (2)	0.37851 (12)	0.0339 (7)	
C1A	0.5216 (4)	1.1773 (3)	0.13866 (19)	0.0317 (9)	
C2A	0.6552 (4)	1.1773 (3)	0.13903 (18)	0.0304 (9)	
C3A	0.8586 (4)	1.2031 (4)	0.1962 (2)	0.0435 (11)	
H3A1	0.9113	1.2468	0.1748	0.052*	
H3A2	0.8636	1.1414	0.1779	0.052*	
C4A	0.9142 (4)	1.2359 (4)	0.2608 (2)	0.0452 (12)	
H4A1	1.0121	1.2532	0.2640	0.054*	
H4A2	0.8920	1.2907	0.2812	0.054*	
C5A	0.8937 (4)	1.1889 (3)	0.34887 (19)	0.0396 (10)	
H5A1	0.8754	1.2461	0.3674	0.047*	
H5A2	0.9901	1.2018	0.3561	0.047*	
C6A	0.8145 (4)	1.1110 (3)	0.37545 (19)	0.0375 (10)	
H6A1	0.8134	1.0506	0.3507	0.045*	
H6A2	0.8559	1.1208	0.4150	0.045*	
C7A	0.5869 (5)	1.0409 (3)	0.39693 (16)	0.0320 (9)	
C8A	0.4550 (4)	1.0409 (3)	0.39693 (16)	0.0313 (9)	
C9A	0.2965 (5)	1.1112 (3)	0.37474 (19)	0.0382 (10)	
H9A1	0.2646	1.1207	0.4141	0.046*	
H9A2	0.2373	1.0508	0.3497	0.046*	
C10A	0.2957 (5)	1.1893 (3)	0.3483 (2)	0.0404 (11)	
H10G	0.2121	1.2022	0.3551	0.049*	
H10H	0.3711	1.2463	0.3670	0.049*	
C11A	0.3212 (5)	1.2358 (4)	0.2599 (2)	0.0455 (12)	
H11A	0.3980	1.2909	0.2801	0.055*	
H11B	0.2403	1.2527	0.2628	0.055*	
C12A	0.3435 (5)	1.2024 (4)	0.1950 (2)	0.0438 (11)	
H12A	0.2769	1.1405	0.1767	0.053*	
H12B	0.3345	1.2459	0.1734	0.053*	
C13A	0.7080 (4)	1.1548 (3)	0.08491 (18)	0.0311 (9)	
H13A	0.7948	1.1515	0.0850	0.037*	
C14A	0.6355 (4)	1.1370 (3)	0.03055 (18)	0.0299 (9)	
C15A	0.5010 (4)	1.1365 (3)	0.03027 (18)	0.0296 (9)	
C16A	0.4464 (4)	1.1551 (3)	0.08463 (18)	0.0306 (9)	
H16A	0.3567	1.1525	0.0846	0.037*	
C17A	0.6171 (5)	0.9720 (3)	0.41512 (17)	0.0372 (10)	
H17A	0.7059	0.9725	0.4156	0.045*	
C18A	0.5160 (5)	0.9009 (3)	0.43301 (17)	0.0376 (10)	
C19A	0.3853 (5)	0.9012 (3)	0.43272 (17)	0.0379 (10)	
C20A	0.3553 (5)	0.9719 (3)	0.41488 (16)	0.0346 (10)	
H20A	0.2671	0.9725	0.4151	0.042*	
P1A	0.19646 (11)	0.39244 (7)	0.14549 (5)	0.0278 (2)	
F1A	0.1875 (3)	0.37509 (17)	0.21079 (11)	0.0372 (6)	
F2A	0.0449 (2)	0.39352 (18)	0.14514 (11)	0.0392 (6)	
F3A	0.2524 (3)	0.50461 (16)	0.17484 (10)	0.0368 (6)	
F4A	0.2050 (3)	0.40926 (17)	0.08006 (10)	0.0365 (6)	
F5A	0.3491 (2)	0.39321 (18)	0.14568 (11)	0.0402 (6)	
F6A	0.1401 (3)	0.28038 (16)	0.11634 (11)	0.0390 (6)	
P1B	0.0000	1.0000	0.5000	0.0398 (4)	
F1B	−0.0190 (4)	0.9672 (3)	0.42946 (13)	0.0703 (9)	
F22B	0.1506 (9)	1.0019 (16)	0.4994 (9)	0.061 (5)	0.30
F32B	−0.046 (2)	0.8950 (7)	0.5037 (11)	0.062 (5)	0.30
F23B	0.1154 (13)	0.9564 (11)	0.4983 (8)	0.080 (4)	0.35
F33B	−0.1021 (11)	0.9003 (6)	0.5006 (6)	0.059 (3)	0.35
F21B	0.1585 (8)	1.0443 (11)	0.5000 (10)	0.079 (5)	0.35
F31B	0.0081 (15)	0.9019 (7)	0.5011 (9)	0.067 (4)	0.35
P1C	1.0000	1.0000	0.0000	0.0819 (9)	
F11C	1.1315 (7)	1.0416 (6)	0.0452 (4)	0.091 (2)	0.70
F21C	0.9550 (12)	0.9040 (4)	0.0199 (4)	0.088 (2)	0.70
F31C	0.9107 (4)	1.0365 (3)	0.0469 (3)	0.0743 (16)	0.70
F12C	1.1046 (19)	1.0121 (15)	0.0538 (9)	0.100 (4)	0.30
F22C	0.934 (3)	0.8909 (7)	−0.0044 (12)	0.117 (7)	0.30
F32C	0.9967 (16)	0.9995 (10)	0.0678 (4)	0.109 (4)	0.30
C1AN	0.9134 (9)	0.8276 (9)	0.1387 (4)	0.055 (3)	0.50
H1A1	0.9349	0.8854	0.1698	0.082*	0.50
H1A2	0.8399	0.8219	0.1117	0.082*	0.50
H1A3	0.9915	0.8279	0.1174	0.082*	0.50
C2AN	0.8752 (10)	0.7509 (9)	0.1639 (5)	0.054 (3)	0.50
N1AN	0.8448 (10)	0.6893 (9)	0.1857 (5)	0.077 (3)	0.50
C1BN	0.703 (2)	0.4071 (14)	0.1200 (8)	0.146 (8)*	0.75
H1B3	0.7262	0.4593	0.1552	0.218*	0.75
H1B4	0.7608	0.3709	0.1210	0.218*	0.75
H1B5	0.6102	0.3679	0.1187	0.218*	0.75
C2BN	0.7218 (18)	0.4410 (10)	0.0692 (6)	0.112 (4)*	0.75
N1BN	0.7401 (10)	0.4796 (7)	0.0313 (5)	0.105 (3)*	0.75
C1CN	0.697 (3)	0.3958 (11)	0.0953 (6)	0.044 (5)	0.25
H1C1	0.7124	0.4431	0.0741	0.065*	0.25
H1C2	0.6053	0.3528	0.0855	0.065*	0.25
H1C3	0.7583	0.3619	0.0841	0.065*	0.25
C2CN	0.720 (2)	0.4395 (14)	0.1575 (6)	0.039 (4)	0.25
N1CN	0.7334 (18)	0.4674 (16)	0.2089 (6)	0.064 (5)	0.25
O2W	0.8088 (13)	0.6125 (9)	0.4157 (6)	0.043 (3)*	0.25
H2WA	0.762 (12)	0.647 (9)	0.432 (7)	0.041 (9)*	0.25
H2WB	0.890 (6)	0.646 (9)	0.433 (7)	0.041 (9)*	0.25
O3W	0.014 (2)	0.0323 (16)	0.2510 (11)	0.093 (6)*	0.25
H3WA	−0.040 (16)	0.064 (13)	0.252 (13)	0.092 (10)*	0.25
H3WB	0.092 (9)	0.077 (11)	0.254 (13)	0.092 (10)*	0.25
O1W	0.7268 (9)	0.4509 (5)	0.3262 (4)	0.068 (2)	0.50
H1WA	0.677 (7)	0.3972 (13)	0.332 (4)	0.063 (6)*	0.50
H1WB	0.739 (11)	0.433 (5)	0.2886 (15)	0.061 (6)*	0.50
O1M	0.6277 (4)	0.2579 (2)	0.31771 (14)	0.0453 (8)	
H1MA	0.607 (4)	0.211 (2)	0.333 (2)	0.044 (5)*	
H1MB	0.702 (3)	0.257 (3)	0.302 (2)	0.049 (5)*	

### Atomic displacement parameters (Å^2^)

**Table:**

	*U*^11^	*U*^22^	*U*^33^	*U*^12^	*U*^13^	*U*^23^
Rh1	0.02500 (18)	0.02144 (18)	0.01569 (17)	0.00805 (12)	−0.00344 (12)	0.00066 (12)
C1C	0.058 (3)	0.041 (3)	0.031 (2)	0.032 (2)	0.003 (2)	−0.0012 (19)
C2C	0.043 (2)	0.046 (3)	0.0172 (18)	0.021 (2)	0.0062 (18)	0.0013 (18)
C3C	0.068 (4)	0.143 (7)	0.043 (3)	0.055 (4)	0.021 (3)	0.054 (4)
C4C	0.060 (3)	0.148 (7)	0.040 (3)	0.047 (4)	0.008 (3)	0.056 (4)
C5C	0.032 (2)	0.047 (3)	0.0179 (18)	0.0153 (19)	−0.0089 (17)	0.0022 (17)
C6C	0.038 (2)	0.040 (3)	0.032 (2)	0.002 (2)	−0.0161 (19)	−0.0012 (19)
C7C	0.064 (4)	0.035 (3)	0.120 (6)	0.009 (3)	−0.038 (4)	−0.032 (3)
C8C	0.072 (4)	0.032 (3)	0.128 (6)	0.017 (3)	0.013 (4)	−0.027 (3)
N1B	0.0321 (18)	0.0330 (18)	0.0203 (16)	0.0150 (14)	−0.0028 (14)	0.0042 (14)
N2B	0.0285 (17)	0.0281 (18)	0.0261 (17)	0.0104 (14)	−0.0047 (14)	0.0015 (14)
N3B	0.0254 (16)	0.0331 (18)	0.0235 (16)	0.0084 (14)	−0.0041 (14)	0.0050 (14)
N4B	0.0277 (17)	0.0289 (18)	0.0239 (16)	0.0092 (14)	0.0000 (14)	0.0014 (14)
C1B	0.037 (2)	0.031 (2)	0.029 (2)	0.0174 (18)	0.0014 (18)	0.0083 (17)
C2B	0.029 (2)	0.049 (3)	0.025 (2)	0.0188 (19)	−0.0056 (17)	0.0055 (19)
C3B	0.031 (2)	0.038 (2)	0.027 (2)	0.0100 (18)	−0.0098 (18)	−0.0006 (18)
C4B	0.0228 (18)	0.026 (2)	0.0224 (18)	0.0102 (15)	−0.0001 (15)	0.0008 (15)
C5B	0.039 (2)	0.028 (2)	0.042 (2)	0.0070 (18)	−0.004 (2)	0.0079 (19)
C6B	0.030 (2)	0.031 (2)	0.027 (2)	0.0073 (17)	−0.0004 (17)	0.0065 (17)
C7B	0.034 (2)	0.041 (2)	0.028 (2)	0.0178 (19)	0.0007 (18)	0.0023 (18)
C8B	0.027 (2)	0.049 (3)	0.0229 (19)	0.0155 (19)	0.0049 (17)	0.0045 (18)
C9B	0.0226 (18)	0.0231 (19)	0.0220 (18)	0.0068 (15)	−0.0050 (15)	0.0018 (15)
C10B	0.044 (2)	0.028 (2)	0.043 (2)	0.0135 (19)	0.006 (2)	0.0092 (19)
Rh2	0.0490 (2)	0.0290 (2)	0.0288 (2)	0.01073 (16)	−0.00532 (17)	−0.00377 (15)
C1E	0.070 (4)	0.035 (3)	0.053 (3)	0.021 (2)	0.001 (3)	0.006 (2)
C2E	0.071 (3)	0.041 (3)	0.036 (2)	0.024 (2)	−0.003 (2)	0.008 (2)
C3E	0.110 (6)	0.136 (7)	0.042 (3)	0.079 (6)	−0.002 (4)	0.013 (4)
C4E	0.066 (4)	0.136 (7)	0.043 (3)	0.016 (4)	−0.006 (3)	0.015 (4)
C5E	0.061 (3)	0.042 (3)	0.030 (2)	0.003 (2)	0.006 (2)	0.007 (2)
C6E	0.052 (3)	0.037 (3)	0.054 (3)	0.005 (2)	−0.004 (2)	0.006 (2)
C7E	0.061 (4)	0.035 (3)	0.178 (9)	0.005 (3)	−0.011 (5)	0.032 (4)
C8E	0.078 (5)	0.034 (3)	0.178 (9)	0.021 (3)	0.027 (5)	0.028 (4)
N1D	0.049 (2)	0.035 (2)	0.0308 (19)	0.0139 (17)	−0.0159 (18)	0.0006 (16)
N2D	0.042 (2)	0.042 (2)	0.037 (2)	0.0175 (18)	−0.0125 (18)	−0.0065 (17)
N3D	0.047 (2)	0.034 (2)	0.0329 (19)	0.0126 (17)	0.0077 (17)	0.0019 (16)
N4D	0.037 (2)	0.043 (2)	0.035 (2)	0.0141 (17)	−0.0022 (17)	−0.0045 (17)
C1D	0.074 (3)	0.033 (2)	0.023 (2)	0.014 (2)	−0.014 (2)	0.0012 (18)
C2D	0.036 (2)	0.048 (3)	0.052 (3)	0.015 (2)	−0.011 (2)	0.009 (2)
C3D	0.033 (2)	0.051 (3)	0.055 (3)	0.018 (2)	−0.011 (2)	−0.002 (2)
C4D	0.042 (2)	0.032 (2)	0.027 (2)	0.0130 (19)	−0.0102 (19)	−0.0015 (17)
C5D	0.048 (3)	0.049 (3)	0.041 (3)	0.019 (2)	−0.011 (2)	−0.012 (2)
C6D	0.066 (3)	0.032 (2)	0.022 (2)	0.012 (2)	0.010 (2)	0.0004 (18)
C7D	0.035 (2)	0.044 (3)	0.052 (3)	0.016 (2)	0.010 (2)	0.007 (2)
C8D	0.034 (2)	0.057 (3)	0.049 (3)	0.021 (2)	0.003 (2)	0.002 (2)
C9D	0.041 (2)	0.033 (2)	0.027 (2)	0.0103 (19)	0.0000 (19)	−0.0016 (18)
C10D	0.045 (3)	0.049 (3)	0.039 (3)	0.017 (2)	−0.008 (2)	−0.013 (2)
O1	0.0407 (16)	0.0329 (16)	0.0259 (14)	0.0113 (13)	−0.0031 (13)	0.0054 (12)
O2	0.0378 (16)	0.0405 (17)	0.0263 (14)	0.0085 (13)	−0.0040 (13)	0.0089 (13)
O3	0.0340 (15)	0.0422 (17)	0.0265 (14)	0.0113 (13)	−0.0012 (13)	0.0115 (13)
O4	0.0386 (16)	0.0404 (17)	0.0267 (14)	0.0180 (13)	0.0045 (13)	0.0121 (13)
O5	0.0503 (18)	0.0416 (17)	0.0241 (14)	0.0225 (15)	0.0032 (13)	0.0092 (13)
O6	0.0415 (16)	0.0339 (16)	0.0258 (14)	0.0140 (13)	−0.0002 (13)	0.0059 (12)
C1A	0.038 (2)	0.031 (2)	0.030 (2)	0.0132 (18)	0.0010 (18)	0.0136 (17)
C2A	0.034 (2)	0.029 (2)	0.029 (2)	0.0093 (17)	0.0016 (18)	0.0133 (17)
C3A	0.030 (2)	0.064 (3)	0.039 (2)	0.009 (2)	0.002 (2)	0.027 (2)
C4A	0.029 (2)	0.054 (3)	0.043 (3)	−0.001 (2)	−0.008 (2)	0.020 (2)
C5A	0.034 (2)	0.046 (3)	0.031 (2)	0.009 (2)	−0.0103 (19)	0.004 (2)
C6A	0.044 (2)	0.045 (3)	0.025 (2)	0.018 (2)	−0.0053 (19)	0.0078 (19)
C7A	0.048 (2)	0.029 (2)	0.0130 (17)	0.0098 (18)	−0.0031 (17)	−0.0010 (15)
C8A	0.047 (2)	0.029 (2)	0.0135 (17)	0.0116 (18)	−0.0010 (17)	−0.0004 (15)
C9A	0.040 (2)	0.048 (3)	0.024 (2)	0.014 (2)	0.0056 (19)	0.0068 (19)
C10A	0.048 (3)	0.047 (3)	0.030 (2)	0.027 (2)	0.004 (2)	0.003 (2)
C11A	0.055 (3)	0.056 (3)	0.044 (3)	0.038 (3)	0.014 (2)	0.021 (2)
C12A	0.049 (3)	0.063 (3)	0.040 (3)	0.037 (2)	0.010 (2)	0.027 (2)
C13A	0.033 (2)	0.032 (2)	0.032 (2)	0.0133 (17)	0.0001 (18)	0.0135 (18)
C14A	0.040 (2)	0.026 (2)	0.0260 (19)	0.0115 (17)	0.0032 (17)	0.0108 (16)
C15A	0.033 (2)	0.027 (2)	0.028 (2)	0.0076 (17)	−0.0023 (17)	0.0097 (16)
C16A	0.029 (2)	0.037 (2)	0.029 (2)	0.0114 (17)	0.0044 (17)	0.0159 (18)
C17A	0.057 (3)	0.034 (2)	0.0153 (18)	0.017 (2)	−0.0090 (19)	−0.0036 (16)
C18A	0.060 (3)	0.027 (2)	0.0170 (18)	0.009 (2)	−0.0047 (19)	−0.0022 (16)
C19A	0.058 (3)	0.029 (2)	0.0163 (18)	0.008 (2)	−0.0001 (19)	−0.0023 (16)
C20A	0.047 (3)	0.032 (2)	0.0140 (17)	0.0071 (19)	0.0001 (18)	−0.0025 (16)
P1A	0.0322 (5)	0.0249 (5)	0.0232 (5)	0.0090 (4)	−0.0024 (4)	0.0027 (4)
F1A	0.0494 (15)	0.0320 (13)	0.0295 (12)	0.0109 (11)	0.0015 (11)	0.0117 (10)
F2A	0.0361 (13)	0.0452 (15)	0.0392 (14)	0.0191 (11)	0.0050 (11)	0.0094 (12)
F3A	0.0531 (15)	0.0251 (12)	0.0273 (12)	0.0085 (11)	−0.0008 (11)	0.0054 (10)
F4A	0.0453 (14)	0.0388 (14)	0.0228 (11)	0.0135 (11)	−0.0026 (10)	0.0057 (10)
F5A	0.0335 (13)	0.0446 (15)	0.0415 (14)	0.0144 (11)	−0.0043 (11)	0.0089 (12)
F6A	0.0446 (14)	0.0252 (12)	0.0385 (14)	0.0086 (11)	−0.0046 (11)	−0.0018 (10)
P1B	0.0355 (9)	0.0525 (11)	0.0317 (8)	0.0184 (8)	−0.0011 (7)	0.0079 (7)
F1B	0.090 (2)	0.082 (2)	0.0338 (16)	0.0303 (19)	−0.0012 (16)	0.0050 (15)
F22B	0.034 (6)	0.088 (11)	0.061 (6)	0.031 (6)	−0.001 (6)	0.008 (11)
F32B	0.055 (10)	0.070 (6)	0.063 (7)	0.021 (6)	−0.011 (10)	0.024 (5)
F23B	0.065 (7)	0.083 (7)	0.078 (6)	0.019 (6)	−0.009 (6)	0.009 (7)
F33B	0.048 (6)	0.073 (6)	0.053 (5)	0.022 (5)	−0.008 (6)	0.009 (4)
F21B	0.068 (7)	0.080 (9)	0.081 (7)	0.030 (6)	0.004 (5)	0.003 (8)
F31B	0.054 (7)	0.076 (6)	0.066 (6)	0.023 (5)	−0.002 (7)	0.010 (5)
P1C	0.0369 (10)	0.0391 (11)	0.153 (3)	0.0145 (9)	−0.0049 (14)	−0.0050 (14)
F11C	0.034 (4)	0.060 (5)	0.158 (6)	0.008 (3)	−0.005 (4)	0.007 (4)
F21C	0.097 (5)	0.043 (3)	0.104 (6)	0.011 (3)	−0.014 (5)	0.006 (3)
F31C	0.0227 (18)	0.057 (3)	0.125 (4)	0.0214 (18)	−0.002 (2)	−0.016 (3)
F12C	0.028 (5)	0.066 (7)	0.174 (7)	0.012 (5)	−0.001 (6)	−0.018 (6)
F22C	0.099 (10)	0.043 (8)	0.164 (14)	0.007 (7)	−0.029 (12)	−0.028 (9)
F32C	0.069 (5)	0.068 (5)	0.150 (6)	0.001 (5)	−0.005 (6)	−0.007 (6)
C1AN	0.031 (5)	0.109 (9)	0.028 (5)	0.040 (5)	0.004 (4)	0.005 (5)
C2AN	0.024 (4)	0.076 (8)	0.061 (7)	0.030 (5)	−0.002 (4)	0.000 (6)
N1AN	0.046 (5)	0.100 (9)	0.073 (7)	0.038 (6)	−0.016 (5)	−0.014 (6)
C1CN	0.087 (13)	0.010 (7)	0.015 (8)	−0.008 (7)	−0.010 (8)	0.005 (6)
C2CN	0.041 (8)	0.053 (9)	0.020 (8)	0.022 (7)	−0.012 (7)	0.000 (7)
N1CN	0.044 (9)	0.104 (13)	0.036 (8)	0.031 (9)	−0.009 (7)	−0.002 (9)
O1W	0.089 (5)	0.045 (4)	0.062 (4)	0.012 (4)	0.003 (4)	0.015 (3)
O1M	0.055 (2)	0.0443 (19)	0.0305 (16)	0.0154 (16)	0.0011 (15)	0.0024 (14)

### Geometric parameters (Å, º)

**Table:**

Rh1—C9B	2.037 (4)	C10D—H10E	0.9600
Rh1—C4B	2.042 (4)	C10D—H10F	0.9600
Rh1—C5C	2.198 (4)	O1—C7A	1.370 (5)
Rh1—C6C	2.203 (4)	O1—C6A	1.438 (5)
Rh1—C1C	2.205 (4)	O2—C4A	1.408 (6)
Rh1—C2C	2.208 (4)	O2—C5A	1.408 (5)
C1C—C2C	1.371 (7)	O3—C2A	1.359 (5)
C1C—C8C	1.495 (8)	O3—C3A	1.412 (5)
C1C—H1C	0.9300	O4—C1A	1.362 (5)
C2C—C3C	1.497 (6)	O4—C12A	1.422 (5)
C2C—H2C	0.9300	O5—C10A	1.407 (5)
C3C—C4C	1.417 (8)	O5—C11A	1.417 (6)
C3C—H3C	0.9300	O6—C8A	1.379 (5)
C4C—C5C	1.503 (7)	O6—C9A	1.440 (5)
C4C—H4C	0.9300	C1A—C16A	1.384 (6)
C5C—C6C	1.354 (7)	C1A—C2A	1.404 (6)
C5C—H5C	0.9300	C2A—C13A	1.389 (6)
C6C—C7C	1.497 (8)	C3A—C4A	1.506 (6)
C6C—H6C	0.9300	C3A—H3A1	0.9700
C7C—C8C	1.402 (9)	C3A—H3A2	0.9700
C7C—H7C	0.9300	C4A—H4A1	0.9700
C8C—H8C	0.9300	C4A—H4A2	0.9700
N1B—C4B	1.350 (5)	C5A—C6A	1.505 (6)
N1B—C2B	1.384 (5)	C5A—H5A1	0.9700
N1B—C1B	1.468 (5)	C5A—H5A2	0.9700
N2B—C4B	1.351 (5)	C6A—H6A1	0.9700
N2B—C3B	1.389 (5)	C6A—H6A2	0.9700
N2B—C5B	1.448 (5)	C7A—C17A	1.378 (6)
N3B—C9B	1.351 (5)	C7A—C8A	1.386 (6)
N3B—C8B	1.383 (5)	C8A—C20A	1.382 (6)
N3B—C6B	1.475 (5)	C9A—C10A	1.503 (7)
N4B—C9B	1.354 (5)	C9A—H9A1	0.9700
N4B—C7B	1.385 (6)	C9A—H9A2	0.9700
N4B—C10B	1.454 (6)	C10A—H10G	0.9700
C1B—C14A	1.515 (6)	C10A—H10H	0.9700
C1B—H1B1	0.9700	C11A—C12A	1.514 (7)
C1B—H1B2	0.9700	C11A—H11A	0.9700
C2B—C3B	1.341 (7)	C11A—H11B	0.9700
C2B—H2B	0.9300	C12A—H12A	0.9700
C3B—H3B	0.9300	C12A—H12B	0.9700
C5B—H5B1	0.9600	C13A—C14A	1.390 (6)
C5B—H5B2	0.9600	C13A—H13A	0.9300
C5B—H5B3	0.9600	C14A—C15A	1.410 (6)
C6B—C15A	1.509 (5)	C15A—C16A	1.395 (6)
C6B—H6B1	0.9700	C16A—H16A	0.9300
C6B—H6B2	0.9700	C17A—C18A	1.409 (7)
C7B—C8B	1.345 (7)	C17A—H17A	0.9300
C7B—H7B	0.9300	C18A—C19A	1.375 (7)
C8B—H8B	0.9300	C19A—C20A	1.399 (6)
C10B—H10A	0.9600	C20A—H20A	0.9300
C10B—H10B	0.9600	P1A—F2A	1.599 (3)
C10B—H10C	0.9600	P1A—F5A	1.600 (3)
Rh2—C4D	2.033 (5)	P1A—F6A	1.606 (3)
Rh2—C9D	2.037 (5)	P1A—F3A	1.608 (3)
Rh2—C1E	2.196 (5)	P1A—F4A	1.608 (2)
Rh2—C6E	2.196 (5)	P1A—F1A	1.608 (2)
Rh2—C2E	2.204 (5)	P1B—F31B^i^	1.570 (7)
Rh2—C5E	2.214 (5)	P1B—F31B	1.570 (7)
C1E—C2E	1.383 (7)	P1B—F33B^i^	1.572 (7)
C1E—C8E	1.508 (8)	P1B—F33B	1.572 (7)
C1E—H1E	0.9300	P1B—F22B^i^	1.573 (7)
C2E—C3E	1.501 (9)	P1B—F22B	1.573 (7)
C2E—H2E	0.9300	P1B—F23B	1.573 (7)
C3E—C4E	1.407 (10)	P1B—F23B^i^	1.573 (7)
C3E—H3E1	0.9700	P1B—F32B	1.574 (7)
C3E—H3E2	0.9700	P1B—F32B^i^	1.574 (7)
C4E—C5E	1.507 (8)	P1B—F21B^i^	1.576 (7)
C4E—H4E1	0.9700	P1B—F21B	1.576 (7)
C4E—H4E2	0.9700	P1C—F31C	1.565 (4)
C5E—C6E	1.396 (7)	P1C—F31C^ii^	1.565 (4)
C5E—H5E	0.9300	P1C—F11C^ii^	1.576 (5)
C6E—C7E	1.502 (8)	P1C—F11C	1.576 (5)
C6E—H6E	0.9300	P1C—F32C^ii^	1.581 (8)
C7E—C8E	1.420 (10)	P1C—F32C	1.581 (8)
C7E—H7E	0.9300	P1C—F22C	1.587 (8)
C8E—H8E	0.9300	P1C—F22C^ii^	1.587 (8)
N1D—C4D	1.351 (6)	P1C—F12C	1.595 (8)
N1D—C2D	1.407 (7)	P1C—F12C^ii^	1.595 (8)
N1D—C1D	1.456 (6)	P1C—F21C	1.603 (5)
N2D—C4D	1.358 (6)	P1C—F21C^ii^	1.603 (5)
N2D—C3D	1.403 (7)	F12C—F32C	1.15 (3)
N2D—C5D	1.474 (6)	C1AN—C2AN	1.407 (13)
N3D—C9D	1.359 (6)	C1AN—H1A1	0.9600
N3D—C7D	1.385 (6)	C1AN—H1A2	0.9600
N3D—C6D	1.468 (6)	C1AN—H1A3	0.9600
N4D—C9D	1.355 (6)	C2AN—N1AN	1.152 (12)
N4D—C8D	1.399 (6)	C1BN—C2BN	1.404 (14)
N4D—C10D	1.466 (6)	C1BN—H1B3	0.9600
C1D—C18A	1.516 (6)	C1BN—H1B4	0.9600
C1D—H1D1	0.9700	C1BN—H1B5	0.9600
C1D—H1D2	0.9700	C2BN—N1BN	1.171 (12)
C2D—C3D	1.329 (7)	C1CN—C2CN	1.417 (14)
C2D—H2D	0.9300	C1CN—H1C1	0.9600
C3D—H3D	0.9300	C1CN—H1C2	0.9600
C5D—H5D1	0.9600	C1CN—H1C3	0.9600
C5D—H5D2	0.9600	C2CN—N1CN	1.156 (13)
C5D—H5D3	0.9600	O2W—H2WA	0.867 (10)
C6D—C19A	1.528 (7)	O2W—H2WB	0.867 (11)
C6D—H6D1	0.9700	O3W—H3WA	0.871 (11)
C6D—H6D2	0.9700	O3W—H3WB	0.870 (11)
C7D—C8D	1.341 (7)	O1W—H1WA	0.870 (10)
C7D—H7D	0.9300	O1W—H1WB	0.871 (10)
C8D—H8D	0.9300	O1M—H1MA	0.863 (10)
C10D—H10D	0.9600	O1M—H1MB	0.860 (10)
			
C9B—Rh1—C4B	92.09 (15)	O3—C3A—C4A	108.0 (4)
C9B—Rh1—C5C	90.57 (15)	O3—C3A—H3A1	110.1
C4B—Rh1—C5C	162.87 (17)	C4A—C3A—H3A1	110.1
C9B—Rh1—C6C	90.60 (17)	O3—C3A—H3A2	110.1
C4B—Rh1—C6C	160.89 (17)	C4A—C3A—H3A2	110.1
C5C—Rh1—C6C	35.85 (17)	H3A1—C3A—H3A2	108.4
C9B—Rh1—C1C	160.62 (18)	O2—C4A—C3A	108.2 (4)
C4B—Rh1—C1C	90.47 (17)	O2—C4A—H4A1	110.1
C5C—Rh1—C1C	92.62 (17)	C3A—C4A—H4A1	110.1
C6C—Rh1—C1C	80.97 (19)	O2—C4A—H4A2	110.1
C9B—Rh1—C2C	162.78 (16)	C3A—C4A—H4A2	110.1
C4B—Rh1—C2C	90.74 (16)	H4A1—C4A—H4A2	108.4
C5C—Rh1—C2C	81.94 (16)	O2—C5A—C6A	109.3 (4)
C6C—Rh1—C2C	92.27 (18)	O2—C5A—H5A1	109.8
C1C—Rh1—C2C	36.21 (18)	C6A—C5A—H5A1	109.8
C2C—C1C—C8C	125.2 (5)	O2—C5A—H5A2	109.8
C2C—C1C—Rh1	72.0 (2)	C6A—C5A—H5A2	109.8
C8C—C1C—Rh1	108.7 (4)	H5A1—C5A—H5A2	108.3
C2C—C1C—H1C	117.4	O1—C6A—C5A	107.6 (3)
C8C—C1C—H1C	117.4	O1—C6A—H6A1	110.2
Rh1—C1C—H1C	89.2	C5A—C6A—H6A1	110.2
C1C—C2C—C3C	124.7 (5)	O1—C6A—H6A2	110.2
C1C—C2C—Rh1	71.8 (3)	C5A—C6A—H6A2	110.2
C3C—C2C—Rh1	107.7 (3)	H6A1—C6A—H6A2	108.5
C1C—C2C—H2C	117.6	O1—C7A—C17A	125.0 (4)
C3C—C2C—H2C	117.6	O1—C7A—C8A	115.6 (4)
Rh1—C2C—H2C	90.5	C17A—C7A—C8A	119.4 (4)
C4C—C3C—C2C	119.6 (5)	O6—C8A—C20A	124.6 (4)
C4C—C3C—H3C	120.2	O6—C8A—C7A	115.6 (4)
C2C—C3C—H3C	120.2	C20A—C8A—C7A	119.9 (4)
C3C—C4C—C5C	119.2 (4)	O6—C9A—C10A	107.5 (4)
C3C—C4C—H4C	120.4	O6—C9A—H9A1	110.2
C5C—C4C—H4C	120.4	C10A—C9A—H9A1	110.2
C6C—C5C—C4C	124.3 (5)	O6—C9A—H9A2	110.2
C6C—C5C—Rh1	72.3 (2)	C10A—C9A—H9A2	110.2
C4C—C5C—Rh1	108.0 (3)	H9A1—C9A—H9A2	108.5
C6C—C5C—H5C	117.9	O5—C10A—C9A	108.8 (4)
C4C—C5C—H5C	117.9	O5—C10A—H10G	109.9
Rh1—C5C—H5C	89.7	C9A—C10A—H10G	109.9
C5C—C6C—C7C	126.1 (6)	O5—C10A—H10H	109.9
C5C—C6C—Rh1	71.9 (2)	C9A—C10A—H10H	109.9
C7C—C6C—Rh1	108.4 (3)	H10G—C10A—H10H	108.3
C5C—C6C—H6C	116.9	O5—C11A—C12A	108.2 (4)
C7C—C6C—H6C	116.9	O5—C11A—H11A	110.1
Rh1—C6C—H6C	89.7	C12A—C11A—H11A	110.1
C8C—C7C—C6C	119.5 (5)	O5—C11A—H11B	110.1
C8C—C7C—H7C	120.3	C12A—C11A—H11B	110.1
C6C—C7C—H7C	120.3	H11A—C11A—H11B	108.4
C7C—C8C—C1C	118.9 (5)	O4—C12A—C11A	107.4 (4)
C7C—C8C—H8C	120.5	O4—C12A—H12A	110.2
C1C—C8C—H8C	120.5	C11A—C12A—H12A	110.2
C4B—N1B—C2B	110.7 (3)	O4—C12A—H12B	110.2
C4B—N1B—C1B	124.5 (3)	C11A—C12A—H12B	110.2
C2B—N1B—C1B	124.8 (3)	H12A—C12A—H12B	108.5
C4B—N2B—C3B	110.7 (3)	C2A—C13A—C14A	122.2 (4)
C4B—N2B—C5B	125.4 (3)	C2A—C13A—H13A	118.9
C3B—N2B—C5B	123.9 (4)	C14A—C13A—H13A	118.9
C9B—N3B—C8B	111.2 (3)	C13A—C14A—C15A	118.8 (4)
C9B—N3B—C6B	124.6 (3)	C13A—C14A—C1B	117.9 (4)
C8B—N3B—C6B	124.2 (3)	C15A—C14A—C1B	123.3 (3)
C9B—N4B—C7B	111.1 (3)	C16A—C15A—C14A	118.7 (4)
C9B—N4B—C10B	124.7 (3)	C16A—C15A—C6B	117.8 (4)
C7B—N4B—C10B	124.2 (4)	C14A—C15A—C6B	123.4 (4)
N1B—C1B—C14A	111.6 (3)	C1A—C16A—C15A	122.0 (4)
N1B—C1B—H1B1	109.3	C1A—C16A—H16A	119.0
C14A—C1B—H1B1	109.3	C15A—C16A—H16A	119.0
N1B—C1B—H1B2	109.3	C7A—C17A—C18A	121.1 (5)
C14A—C1B—H1B2	109.3	C7A—C17A—H17A	119.4
H1B1—C1B—H1B2	108.0	C18A—C17A—H17A	119.4
C3B—C2B—N1B	107.1 (3)	C19A—C18A—C17A	119.1 (4)
C3B—C2B—H2B	126.5	C19A—C18A—C1D	124.3 (4)
N1B—C2B—H2B	126.5	C17A—C18A—C1D	116.6 (4)
C2B—C3B—N2B	106.5 (4)	C18A—C19A—C20A	119.6 (4)
C2B—C3B—H3B	126.7	C18A—C19A—C6D	123.7 (4)
N2B—C3B—H3B	126.7	C20A—C19A—C6D	116.7 (4)
N1B—C4B—N2B	105.0 (3)	C8A—C20A—C19A	120.9 (4)
N1B—C4B—Rh1	127.1 (3)	C8A—C20A—H20A	119.6
N2B—C4B—Rh1	127.7 (3)	C19A—C20A—H20A	119.6
N2B—C5B—H5B1	109.5	F2A—P1A—F5A	178.98 (15)
N2B—C5B—H5B2	109.5	F2A—P1A—F6A	90.39 (14)
H5B1—C5B—H5B2	109.5	F5A—P1A—F6A	90.49 (14)
N2B—C5B—H5B3	109.5	F2A—P1A—F3A	89.45 (15)
H5B1—C5B—H5B3	109.5	F5A—P1A—F3A	89.67 (15)
H5B2—C5B—H5B3	109.5	F6A—P1A—F3A	179.78 (18)
N3B—C6B—C15A	111.6 (3)	F2A—P1A—F4A	89.73 (14)
N3B—C6B—H6B1	109.3	F5A—P1A—F4A	89.75 (14)
C15A—C6B—H6B1	109.3	F6A—P1A—F4A	90.21 (14)
N3B—C6B—H6B2	109.3	F3A—P1A—F4A	89.94 (13)
C15A—C6B—H6B2	109.3	F2A—P1A—F1A	90.22 (14)
H6B1—C6B—H6B2	108.0	F5A—P1A—F1A	90.31 (14)
C8B—C7B—N4B	106.6 (4)	F6A—P1A—F1A	89.52 (14)
C8B—C7B—H7B	126.7	F3A—P1A—F1A	90.33 (13)
N4B—C7B—H7B	126.7	F4A—P1A—F1A	179.73 (17)
C7B—C8B—N3B	106.7 (4)	F31B^i^—P1B—F31B	179.997 (8)
C7B—C8B—H8B	126.7	F31B—P1B—F33B^i^	137.1 (6)
N3B—C8B—H8B	126.7	F31B^i^—P1B—F33B	137.1 (6)
N3B—C9B—N4B	104.4 (3)	F33B^i^—P1B—F33B	179.999 (3)
N3B—C9B—Rh1	127.1 (3)	F31B^i^—P1B—F22B^i^	68.8 (9)
N4B—C9B—Rh1	128.3 (3)	F31B—P1B—F22B^i^	111.2 (9)
N4B—C10B—H10A	109.5	F33B^i^—P1B—F22B^i^	111.7 (8)
N4B—C10B—H10B	109.5	F33B—P1B—F22B^i^	68.3 (8)
H10A—C10B—H10B	109.5	F31B^i^—P1B—F22B	111.2 (9)
N4B—C10B—H10C	109.5	F31B—P1B—F22B	68.8 (9)
H10A—C10B—H10C	109.5	F33B^i^—P1B—F22B	68.3 (8)
H10B—C10B—H10C	109.5	F33B—P1B—F22B	111.7 (8)
C4D—Rh2—C9D	93.91 (18)	F22B^i^—P1B—F22B	180.0 (17)
C4D—Rh2—C1E	90.3 (2)	F31B^i^—P1B—F23B	136.0 (7)
C9D—Rh2—C1E	163.54 (19)	F33B^i^—P1B—F23B	93.1 (7)
C4D—Rh2—C6E	163.53 (19)	F33B—P1B—F23B	86.9 (7)
C9D—Rh2—C6E	90.5 (2)	F22B^i^—P1B—F23B	155.2 (7)
C1E—Rh2—C6E	81.3 (2)	F31B—P1B—F23B^i^	136.0 (7)
C4D—Rh2—C2E	89.0 (2)	F33B^i^—P1B—F23B^i^	86.9 (7)
C9D—Rh2—C2E	159.12 (18)	F33B—P1B—F23B^i^	93.1 (7)
C1E—Rh2—C2E	36.66 (19)	F22B—P1B—F23B^i^	155.2 (7)
C6E—Rh2—C2E	92.5 (2)	F23B—P1B—F23B^i^	179.996 (9)
C4D—Rh2—C5E	158.85 (18)	F31B^i^—P1B—F32B	159.8 (8)
C9D—Rh2—C5E	88.8 (2)	F33B^i^—P1B—F32B	157.0 (6)
C1E—Rh2—C5E	93.1 (2)	F22B^i^—P1B—F32B	91.1 (10)
C6E—Rh2—C5E	36.91 (19)	F22B—P1B—F32B	88.9 (10)
C2E—Rh2—C5E	81.4 (2)	F23B—P1B—F32B	64.2 (8)
C2E—C1E—C8E	125.6 (6)	F23B^i^—P1B—F32B	115.8 (9)
C2E—C1E—Rh2	72.0 (3)	F31B—P1B—F32B^i^	159.8 (8)
C8E—C1E—Rh2	108.9 (4)	F33B—P1B—F32B^i^	157.0 (6)
C2E—C1E—H1E	117.2	F22B^i^—P1B—F32B^i^	88.9 (10)
C8E—C1E—H1E	117.2	F22B—P1B—F32B^i^	91.1 (10)
Rh2—C1E—H1E	89.1	F23B—P1B—F32B^i^	115.8 (9)
C1E—C2E—C3E	125.7 (6)	F23B^i^—P1B—F32B^i^	64.2 (8)
C1E—C2E—Rh2	71.4 (3)	F32B—P1B—F32B^i^	179.999 (8)
C3E—C2E—Rh2	108.7 (4)	F31B^i^—P1B—F21B^i^	92.0 (8)
C1E—C2E—H2E	117.2	F31B—P1B—F21B^i^	88.0 (8)
C3E—C2E—H2E	117.2	F33B^i^—P1B—F21B^i^	135.0 (7)
Rh2—C2E—H2E	89.9	F33B—P1B—F21B^i^	45.0 (7)
C4E—C3E—C2E	118.9 (6)	F22B—P1B—F21B^i^	156.7 (8)
C4E—C3E—H3E1	107.6	F23B—P1B—F21B^i^	131.9 (8)
C2E—C3E—H3E1	107.6	F23B^i^—P1B—F21B^i^	48.1 (8)
C4E—C3E—H3E2	107.6	F32B—P1B—F21B^i^	67.9 (9)
C2E—C3E—H3E2	107.6	F32B^i^—P1B—F21B^i^	112.1 (9)
H3E1—C3E—H3E2	107.0	F31B^i^—P1B—F21B	88.0 (8)
C3E—C4E—C5E	119.9 (6)	F31B—P1B—F21B	92.0 (8)
C3E—C4E—H4E1	107.4	F33B^i^—P1B—F21B	45.0 (7)
C5E—C4E—H4E1	107.4	F33B—P1B—F21B	135.0 (7)
C3E—C4E—H4E2	107.4	F22B^i^—P1B—F21B	156.7 (8)
C5E—C4E—H4E2	107.4	F23B—P1B—F21B	48.1 (8)
H4E1—C4E—H4E2	106.9	F23B^i^—P1B—F21B	131.9 (8)
C6E—C5E—C4E	124.4 (6)	F32B—P1B—F21B	112.1 (9)
C6E—C5E—Rh2	70.9 (3)	F32B^i^—P1B—F21B	67.9 (9)
C4E—C5E—Rh2	107.8 (4)	F21B^i^—P1B—F21B	179.998 (2)
C6E—C5E—H5E	117.8	F31C—P1C—F31C^ii^	180.0 (3)
C4E—C5E—H5E	117.8	F31C—P1C—F11C^ii^	86.0 (5)
Rh2—C5E—H5E	91.4	F31C^ii^—P1C—F11C^ii^	94.0 (4)
C5E—C6E—C7E	125.6 (6)	F31C—P1C—F11C	94.0 (4)
C5E—C6E—Rh2	72.2 (3)	F31C^ii^—P1C—F11C	86.0 (5)
C7E—C6E—Rh2	109.2 (4)	F11C^ii^—P1C—F11C	179.998 (2)
C5E—C6E—H6E	117.2	F31C—P1C—F32C^ii^	128.4 (6)
C7E—C6E—H6E	117.2	F31C^ii^—P1C—F32C^ii^	51.6 (6)
Rh2—C6E—H6E	88.5	F11C^ii^—P1C—F32C^ii^	57.1 (6)
C8E—C7E—C6E	118.7 (5)	F11C—P1C—F32C^ii^	122.9 (6)
C8E—C7E—H7E	120.7	F31C—P1C—F32C	51.6 (6)
C6E—C7E—H7E	120.7	F31C^ii^—P1C—F32C	128.4 (6)
C7E—C8E—C1E	118.5 (5)	F11C^ii^—P1C—F32C	122.9 (6)
C7E—C8E—H8E	120.8	F11C—P1C—F32C	57.1 (6)
C1E—C8E—H8E	120.8	F32C^ii^—P1C—F32C	179.999 (5)
C4D—N1D—C2D	110.8 (4)	F31C—P1C—F22C	98.0 (14)
C4D—N1D—C1D	124.6 (4)	F31C^ii^—P1C—F22C	82.0 (14)
C2D—N1D—C1D	124.6 (4)	F11C^ii^—P1C—F22C	72.3 (10)
C4D—N2D—C3D	111.6 (4)	F11C—P1C—F22C	107.7 (10)
C4D—N2D—C5D	124.9 (4)	F32C^ii^—P1C—F22C	102.4 (11)
C3D—N2D—C5D	123.5 (4)	F32C—P1C—F22C	77.6 (11)
C9D—N3D—C7D	111.3 (4)	F31C—P1C—F22C^ii^	82.0 (14)
C9D—N3D—C6D	123.5 (4)	F31C^ii^—P1C—F22C^ii^	98.0 (14)
C7D—N3D—C6D	125.2 (4)	F11C^ii^—P1C—F22C^ii^	107.7 (10)
C9D—N4D—C8D	111.6 (4)	F11C—P1C—F22C^ii^	72.3 (10)
C9D—N4D—C10D	124.9 (4)	F32C^ii^—P1C—F22C^ii^	77.6 (11)
C8D—N4D—C10D	123.4 (4)	F32C—P1C—F22C^ii^	102.4 (11)
N1D—C1D—C18A	113.3 (3)	F22C—P1C—F22C^ii^	179.997 (9)
N1D—C1D—H1D1	108.9	F31C—P1C—F12C	88.6 (10)
C18A—C1D—H1D1	108.9	F31C^ii^—P1C—F12C	91.4 (10)
N1D—C1D—H1D2	108.9	F11C^ii^—P1C—F12C	160.4 (8)
C18A—C1D—H1D2	108.9	F32C^ii^—P1C—F12C	137.7 (10)
H1D1—C1D—H1D2	107.7	F22C—P1C—F12C	89.8 (12)
C3D—C2D—N1D	107.4 (4)	F22C^ii^—P1C—F12C	90.2 (12)
C3D—C2D—H2D	126.3	F31C—P1C—F12C^ii^	91.4 (10)
N1D—C2D—H2D	126.3	F31C^ii^—P1C—F12C^ii^	88.6 (10)
C2D—C3D—N2D	106.1 (5)	F11C—P1C—F12C^ii^	160.4 (8)
C2D—C3D—H3D	127.0	F32C—P1C—F12C^ii^	137.7 (10)
N2D—C3D—H3D	127.0	F22C—P1C—F12C^ii^	90.2 (12)
N1D—C4D—N2D	104.2 (4)	F22C^ii^—P1C—F12C^ii^	89.8 (12)
N1D—C4D—Rh2	126.1 (3)	F12C—P1C—F12C^ii^	179.998 (4)
N2D—C4D—Rh2	129.0 (3)	F31C—P1C—F21C	88.5 (5)
N2D—C5D—H5D1	109.5	F31C^ii^—P1C—F21C	91.5 (5)
N2D—C5D—H5D2	109.5	F11C^ii^—P1C—F21C	89.5 (4)
H5D1—C5D—H5D2	109.5	F11C—P1C—F21C	90.5 (4)
N2D—C5D—H5D3	109.5	F32C^ii^—P1C—F21C	122.0 (6)
H5D1—C5D—H5D3	109.5	F32C—P1C—F21C	58.0 (6)
H5D2—C5D—H5D3	109.5	F22C^ii^—P1C—F21C	159.6 (9)
N3D—C6D—C19A	112.6 (3)	F12C—P1C—F21C	71.4 (8)
N3D—C6D—H6D1	109.1	F12C^ii^—P1C—F21C	108.6 (8)
C19A—C6D—H6D1	109.1	F31C—P1C—F21C^ii^	91.5 (5)
N3D—C6D—H6D2	109.1	F31C^ii^—P1C—F21C^ii^	88.5 (5)
C19A—C6D—H6D2	109.1	F11C^ii^—P1C—F21C^ii^	90.5 (4)
H6D1—C6D—H6D2	107.8	F11C—P1C—F21C^ii^	89.5 (4)
C8D—C7D—N3D	107.3 (4)	F32C^ii^—P1C—F21C^ii^	58.0 (6)
C8D—C7D—H7D	126.3	F32C—P1C—F21C^ii^	122.0 (6)
N3D—C7D—H7D	126.3	F22C—P1C—F21C^ii^	159.6 (9)
C7D—C8D—N4D	105.9 (4)	F12C—P1C—F21C^ii^	108.6 (8)
C7D—C8D—H8D	127.0	F12C^ii^—P1C—F21C^ii^	71.4 (8)
N4D—C8D—H8D	127.0	F21C—P1C—F21C^ii^	179.998 (3)
N4D—C9D—N3D	103.8 (4)	F32C—F12C—P1C	68.2 (7)
N4D—C9D—Rh2	129.1 (3)	F12C—F32C—P1C	69.5 (7)
N3D—C9D—Rh2	126.3 (3)	C2AN—C1AN—H1A1	109.5
N4D—C10D—H10D	109.5	C2AN—C1AN—H1A2	109.5
N4D—C10D—H10E	109.5	H1A1—C1AN—H1A2	109.5
H10D—C10D—H10E	109.5	C2AN—C1AN—H1A3	109.5
N4D—C10D—H10F	109.5	H1A1—C1AN—H1A3	109.5
H10D—C10D—H10F	109.5	H1A2—C1AN—H1A3	109.5
H10E—C10D—H10F	109.5	N1AN—C2AN—C1AN	178.6 (14)
C7A—O1—C6A	116.5 (3)	N1BN—C2BN—C1BN	172.1 (17)
C4A—O2—C5A	111.9 (3)	C2CN—C1CN—H1C1	109.5
C2A—O3—C3A	117.9 (3)	C2CN—C1CN—H1C2	109.5
C1A—O4—C12A	116.9 (3)	H1C1—C1CN—H1C2	109.5
C10A—O5—C11A	111.7 (4)	C2CN—C1CN—H1C3	109.5
C8A—O6—C9A	116.7 (3)	H1C1—C1CN—H1C3	109.5
O4—C1A—C16A	125.4 (4)	H1C2—C1CN—H1C3	109.5
O4—C1A—C2A	115.2 (3)	N1CN—C2CN—C1CN	174 (2)
C16A—C1A—C2A	119.3 (4)	H2WA—O2W—H2WB	101.5 (16)
O3—C2A—C13A	125.2 (4)	H3WA—O3W—H3WB	100.7 (16)
O3—C2A—C1A	116.0 (4)	H1WA—O1W—H1WB	100.5 (16)
C13A—C2A—C1A	118.8 (4)	H1MA—O1M—H1MB	102.6 (16)

Symmetry codes: (i) −*x*, −*y*+2, −*z*+1; (ii) −*x*+2, −*y*+2, −*z*.

### Hydrogen-bond geometry (Å, º)

**Table:**

*D*—H···*A*	*D*—H	H···*A*	*D*···*A*	*D*—H···*A*
O1*M*—H1*MB*···O2^iii^	0.86 (1)	2.51 (3)	3.226 (5)	141 (3)
O1*M*—H1*MB*···O3^iii^	0.86 (1)	2.50 (4)	3.076 (4)	125 (4)
O1*M*—H1*MA*···O1^iii^	0.86 (1)	2.38 (3)	3.149 (5)	149 (4)
O1*M*—H1*MA*···O6^iii^	0.86 (1)	2.39 (2)	3.138 (5)	145 (4)
O3*W*—H3*WB*···O5^iii^	0.87 (1)	2.21 (9)	3.03 (2)	158 (23)
O3*W*—H3*WA*···O2^iv^	0.87 (1)	2.16 (10)	2.99 (2)	160 (27)
O1*W*—H1*WA*···O1*M*	0.87 (1)	2.00 (1)	2.796 (9)	151 (3)
O1*W*—H1*WB*···N1*CN*	0.87 (1)	2.06 (6)	2.805 (18)	143 (9)

Symmetry codes: (iii) *x*, *y*−1, *z*; (iv) *x*−1, *y*−1, *z*.

## References

[bb1] Dolomanov, O. V., Bourhis, L. J., Gildea, R. J., Howard, J. A. K. & Puschmann, H. (2009). *J. Appl. Cryst.* **42**, 339–341.

[bb2] Farrugia, L. J. (2012). *J* *Appl. Cryst* **45**, 849–854.

[bb3] Kabsch, W. (1993). *J. Appl. Cryst.* **26**, 795–800.

[bb4] Mata, J. A., Chianese, A. R., Miecznikowski, J. R., Poyatos, M., Peris, E., Faller, J. W. & Crabtree, R. H. (2004). *Organometallics*, **23**, 1253–1263.

[bb5] McPhillips, T. M., McPhillips, S. E., Chiu, H.-J., Cohen, A. E., Deacon, A. M., Ellis, P. J., Garman, E., Gonzalez, A., Sauter, N. K., Phizackerley, R. P., Soltis, S. M. & Kuhn, P. (2002). *J. Synchrotron Rad.* **9**, 401–406. 10.1107/s0909049502015170 12409628

[bb6] Riederer, S. K. U., Gigler, P., Högerl, M. P., Herdtweck, E., Bechlars, B., Herrmann, W. A. & Kühn, F. (2010). *Organometallics*, **29**, 5681–5692.

[bb7] Sheldrick, G. M. (2008). *Acta Cryst.* A**64**, 112–122. 10.1107/S0108767307043930 18156677

[bb8] Shrestha, S., Gimbert-Suriñach, C., Bhadbhade, M. & Colbran, S. B. (2011). *Eur. J. Inorg. Chem.* **28**, 4331–4337.

[bb9] Westrip, S. P. (2010). *J. Appl. Cryst.* **43**, 920–925.

